# Validating the screening criteria for bone metastases in treatment-naïve unfavorable intermediate and high-risk prostate cancer - the prevalence and location of bone- and lymph node metastases

**DOI:** 10.1007/s00330-022-08945-7

**Published:** 2022-08-08

**Authors:** Erik Rud, Daniyal Noor, Kristina Flor Galtung, Fredrik Ottosson, Maciej Jacewicz, Eduard Baco, Peter Mæhre Lauritzen

**Affiliations:** 1 Division of Radiology and Nuclear Medicine, Oslo University Hospital, Oslo, Norway; 2 Faculty of Medicine, University of Oslo, Oslo, Norway; 3 Department of Urology, Oslo University Hospital, Oslo, Norway

**Keywords:** Prostatic neoplasm, Prevalence, Lymph nodes, Lymphatic metastases, Magnetic resonance imaging

## Abstract

**Objective:**

The European Association of Urology (EAU) recommends a bone scan for newly diagnosed unfavorable intermediate- and high-risk prostate cancer. We aimed to validate the screening criteria for bone metastases in patients with treatment-naïve prostate cancer.

**Methods:**

This single-center retrospective study included all patients with treatment-naïve unfavorable intermediate- or high-risk prostate cancer. All underwent MRI of the lumbar column (T2Dixon) and pelvis (3DT2w, DWI, and T2 Dixon). The presence and location of lymph node and bone metastases were registered according to risk groups and radiological (rad) T-stage. The risk of lymph node metastases was assessed by odds ratio (OR).

**Results:**

We included 390 patients, of which 68% were high-risk and 32% were unfavorable intermediate-risk. In the high-risk group, the rate of regional- and non-regional lymph node metastases was 11% and 6%, respectively, and the rate of bone metastases was 10%. In the unfavorable intermediate-risk group, the rate of regional- and non-regional lymph node metastases was 4% and 0.8%, respectively, and the rate of bone metastases was 0.8%. Metastases occurred exclusively in the lumbar column in 0.5% of all patients, in the pelvis in 4%, and the pelvis and lumbar column in 3%. All patients with bone metastases had radT3-4, and patients with radT3-4 showed a four-fold increased risk of lymph node metastases (OR 4.48, 95% CI: 2.1–9.5).

**Conclusion:**

Bone metastases were found in 10% with high-risk prostate cancer and 0.8% with unfavorable intermediate-risk. Therefore, we question the recommendation to screen the unfavorable intermediate-risk group for bone metastases.

**Key Points:**

*• The rate of bone metastases was 10% in high-risk patients and 0.8% in the unfavorable intermediate-risk group.*

*• The rate of lymph-node metastases was 17% in high-risk patients and 5% in the unfavorable intermediate-risk group.*

*• No bone metastases were seen in radiologically localized disease.*

## Introduction

The detection of metastases is essential for deciding on appropriate treatment and prognosis in newly diagnosed prostate cancer patients. The current guidelines from the European Association of Urology (EAU) recommend, as a minimum, a bone scan (BS) for the detection of bone metastases and abdominal cross-sectional images for lymph node staging in unfavorable intermediate- and high-risk patients [1]. The combination of biopsy results, PSA, and clinical T-stage defines the risk groups, while radiological T (radT) classification has no formal function.

Metastases from prostate cancer first appear in the pelvis before ascending [2, 3]. Several studies have shown that the pelvis is the most prevalent site of metastases, and solitary bone metastases outside the pelvis occur in only 0–0.3% [4–6]. Moreover, bone metastases are mainly prevalent in high-risk patients, but few modern studies report on metastases in the unfavorable intermediate-risk group. We recently analyzed the routine use of whole-body MRI for detecting bone metastases using the current eligibility criteria defined by the EAU. In that study, we did not reveal any bone metastases in the unfavorable-risk group, and we did not discover bone metastases outside the pelvis without concomitant pelvic metastases [4]. Therefore, our institution now uses MRI of the lumbar column and pelvis as the standard screening method for lymph node- and bone metastases.

No imaging modality is sufficiently accurate for lymph node staging, and pelvic lymph node dissection is still the gold standard [1]. In treatment-naïve patients, the sensitivity for detecting lymph node metastases is 33–100% for PSMA PET-CT [7–10], 19–78% for choline PET/CT, 33–57% for DWI MRI [11–13], and 5–94% for CT [14]. To the best of our knowledge, no studies have evaluated the benefit of nodal imaging according to current risk groups.

MRI, choline PET/CT, and PSMA PET/CT are all superior to BS for detecting bone metastases. In mixed populations of treatment-naïve and recurrent disease, the pooled sensitivities are 91–97% for MRI, 97% for PSMA PET-CT, 87–91% for choline PET-CT, 96% for NaF PET-CT, and 79–86% for BS [15, 16]. However, no “head to head” comparisons between MRI and PSMA PET-CT exist in the case of treatment-naïve patients.

Since most centers experience strained imaging capacity, selecting the appropriate patients at risk is essential. We aimed to validate the EAU screening criteria for bone metastases in patients with treatment naïve prostate cancer. We also assessed if radiological T-classification could predict bone metastases.

## Patients and method

**Fig. 1:**
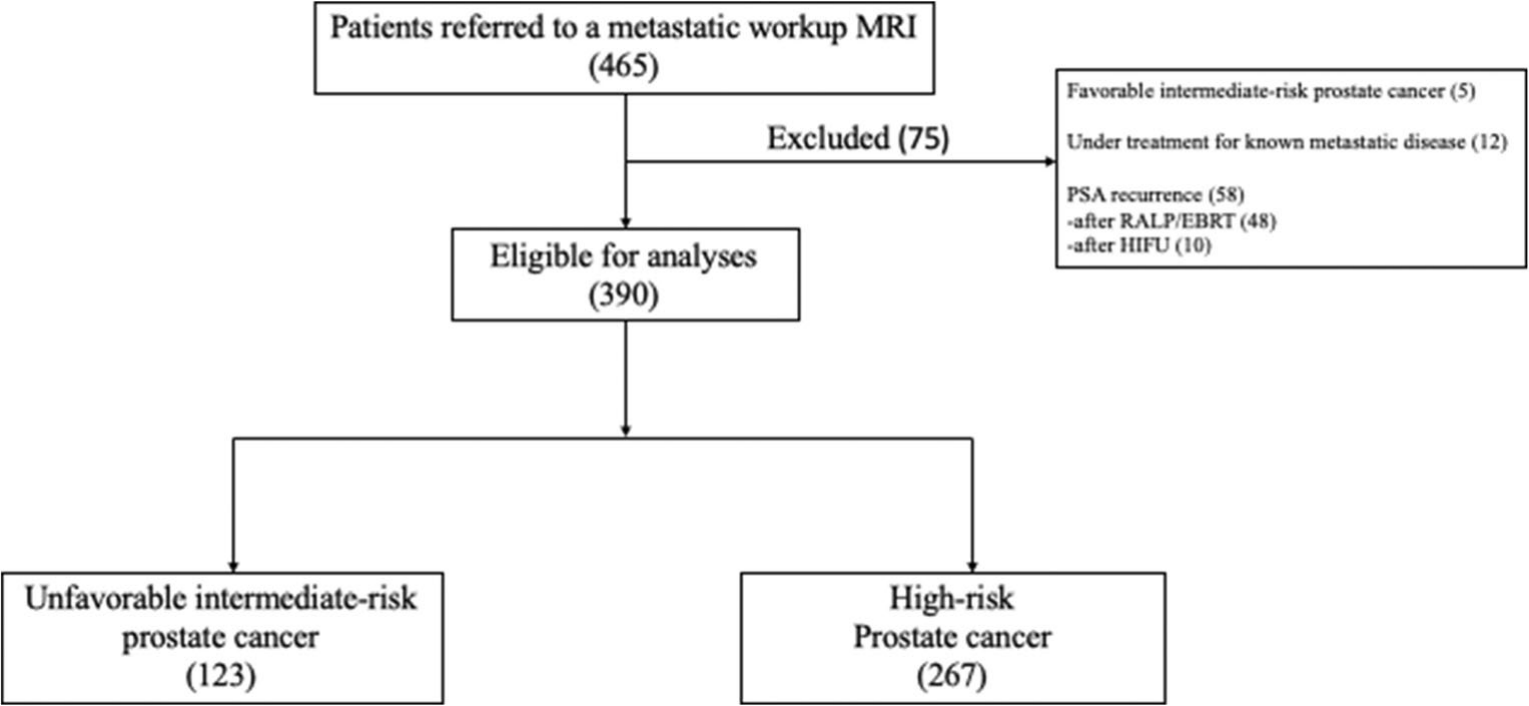
All included and excluded patients

From January 2018 until March 2021, 465 patients underwent a metastatic workup due to prostate cancer. Of these, 390 were treatment naïve and included for analyses in this retrospective study using the original MRI reports (Fig. 1). The Local Data Protection Officer approved the study and issued a waiver of informed consent (20/13111).

### Inclusion criteria

Inclusion criteria were treatment naïve (i) high-risk prostate cancer (International Society of Uropathologist [ISUP] ≥ 4, and/or PSA ≥ 20 ng/ml, and/or cT ≥ 2c) and (ii) unfavorable intermediate-risk prostate cancer (ISUP 3, and PSA < 20 ng/ml and cT< 2c).

### Exclusion criteria

Exclusion criteria were patients with biochemical recurrence after previous treatment or known metastatic disease. Treatment-naïve (i) low-risk prostate cancer (ISUP 1, and PSA < 10 ng/ml, and cT < 2b)- and (ii) favorable intermediate-risk (ISUP ≤ 2 and PSA < 20 ng/ml and cT ≤ 2b).

### Diagnostic workup

The clinical T-stage (cT) was based on digital rectal examination by the referring urologist and classified as localized (cT1-2) or non-localized (cT3-4). All patients underwent a bi-parametric prostatic MRI using 3D T2w images and diffusion-weighted images (DWI) before targeted prostate biopsies with or without systematic biopsies. We used local and external 1.5-T MRI and 3-T MRI. Radiological T-stage (radT) was registered prospectively as localized (radT1-2) or non-localized (radT3-4) according to a structured reporting template at our institution. External prostate MRIs were assessed for radT-stage at weekly multidisciplinary meetings following referral. The criteria for radT3-4 were defined as obliterated rectoprostatic angle, gross bulging of the capsule, or visible invasion of periprostatic structures [17–19]. In addition, indirect signs of extraprostatic extension, i.e., tumor capsule length > 15 mm and apparent diffusion coefficient < 90 × 10^-5 mm^/s^2^, were used, although no absolute cut-off values were defined a priori [20–23].

### Metastatic workup

Based on the biopsy results, PSA, and cT-stage, eligible patients underwent a metastatic workup of the lumbosacral column and pelvis using 1.5-T MRI (Avanto fit, Siemens Healthcare). Applied MRI sequences were axial 3D T2 and DWI of the pelvis and coronal T2 Dixon of the lumbar column and pelvis (Table 1).

**Table 1:** MRI acquisition parameters

Sequence	Region	Plane of acquisition	Time of repetition (ms)	Time of echo (ms)	Slice thickness (mm)	Voxel size/reconstructed (mm × mm × mm)	Field of view (mm × mm)	Scan time (min:sec)
T2_tse	Pelvis	Sagittal	2810	120	3.3	0.78 × 0.78 × 3.3/0.39 × 0.39 × 3.3	200 × 200	2:02
DWI (epi b0_b800)	Pelvis	Transversal	8000	63	5	2.46 × 2.46 × 5/1.23 × 1.23 × 5	320 × 226	3:11
T2_spc_3D	Pelvis	Transversal	1300	103	1	1 × 1 × 1/0.5 × 0.5 × 1	256 × 256	10:20
T2_tse_Dixon fat and water	Pelvis/lumbar spine	Coronal	7200	69	4	0.99 × 0.89 × 4/0.45 × 0.45 × 4	400 × 400	3:02

*tse*, turbo spin echo; *DWI*, diffusion-weighted images. We used the spectral adiabatic inversion recovery (SPAIR) fat suppression technique

The purpose of 3DT2 and DWI of the pelvis was to detect lymph node metastases, and DWI was also used to detect bone metastases when combined with T2 Dixon. We used T2 Dixon of the lumbar column to detect bone metastases and enlarged retroperitoneal lymph nodes. Supplementary sagittal DWI and T2 Dixon were obtained from the complete column in case of equivocal findings.

All MRI readings were performed by consultant radiologists specialized in oncological imaging. Eight radiologists with 5–15 years of experience were involved. All MRIs were classified as negative or positive for lymph node- and bone metastases based on the original report.

#### Lymph node metastases

The definition of lymph node metastases was descriptive based on the original report and at the discretion of the reading radiologist. No rigid criteria were set a priori, although lymph nodes were considered metastatic if the short axis was > 5 mm and hyperintense compared to surrounding tissue on high *b*-value images [24, 25]. Positive nodes were classified as regional if located below the bifurcation of the common iliac artery or non-regional if located above [1]. Equivocal and negative reports were both classified as negative.

#### Bone metastases

The definition of bone metastases was descriptive, based on the original report, and at the discretion of the reading radiologist, unless a biopsy was performed. All available sequences were assessed, and only lesions > 5 mm were considered. On T2-Dixon, lesions were considered metastases if hypointense on the fat sequence compared to skeletal muscle and hyperintense on the water sequence compared to normal marrow and muscle tissue [26–28]. In the case of punctuating high intralesional signals on T2 Dixon-Fat, these lesions were considered benign due to intralesional fat [27]. On DWI, lesions were considered metastases if hyperintense on high *b-*value images compared to normal marrow and muscle tissue and if the corresponding apparent diffusion coefficient (ADC) map demonstrated iso- or slightly higher signal compared to normal marrow [29]. No rigid ADC cut-off values were used, and the evaluation was based on the radiologist’s discretion. DWI of the column was only performed in case of equivocal findings on T2 Dixon.

In case of positive findings, the size, number, and location were registered. All bone metastases ≥ 50 mm were reported as 50mm. In the case of the equivocal conclusions, patients were discussed during multidisciplinary meetings and deemed as negative or positive depending on the overall clinical suspicion.

### Statistics

The rate of metastases with 95% confidence intervals (CIs) was reported in the different risk groups. Median values with interquartile ranges (IQRs) were presented for non-normal distributed continuous variables. Patients were dichotomized into those having metastases or not, and the Mann-Whitney U test assessed any differences in PSA and age. The correlation between cT and radT was assessed by Kappa. Logistic regression with a binary outcome was used to determine the risk of lymph node and bone metastases in radT1-2 vs. radT3-4 and cT1-2 vs. cT3-4. Logistic regression was also used to assess the risk of bone metastases in patients with- and without lymph node metastases. Results were reported as odds ratio (OR) with 95% CI. The significance level was set at *p* < 0.05. All analyses were performed using SPSS Statistics for Mac, version 27 (IBM Corp), and MedCalc Statistical Software, version 15.11.4 (MedCalc Software Ltd.).

## Results

A total of 390 patients were included for analysis, of which 68% (267 out of 390) were high-risk, and 32 % (123 out of 390) were unfavorable intermediate-risk (Fig. 1). Prostate biopsies were not obtained in nine patients due to comorbidities. All of these nine were classified as high-risk due to PI-RADS 5, radT3-4, and PSA > 20 ng/ml (median 75 ng/ml, IQR 27–324, range 22 to 600).

The median age was 73 years (IQR 67–77), and the median PSA was 11 ng/ml (IQR 8–20). Clinical T3-4 was seen in 20% (76 out of 343, missing data in 47) and radT3-4 in 56% (217 out of 389, missing data in 1). MRI downgraded 13% (10 out of 76) of those classified as cT3-4 and upgraded 47% (124 out of 266) of those classified as cT1-2 (Κappa 0.26).

### Lymph node metastases

**Fig. 2:**
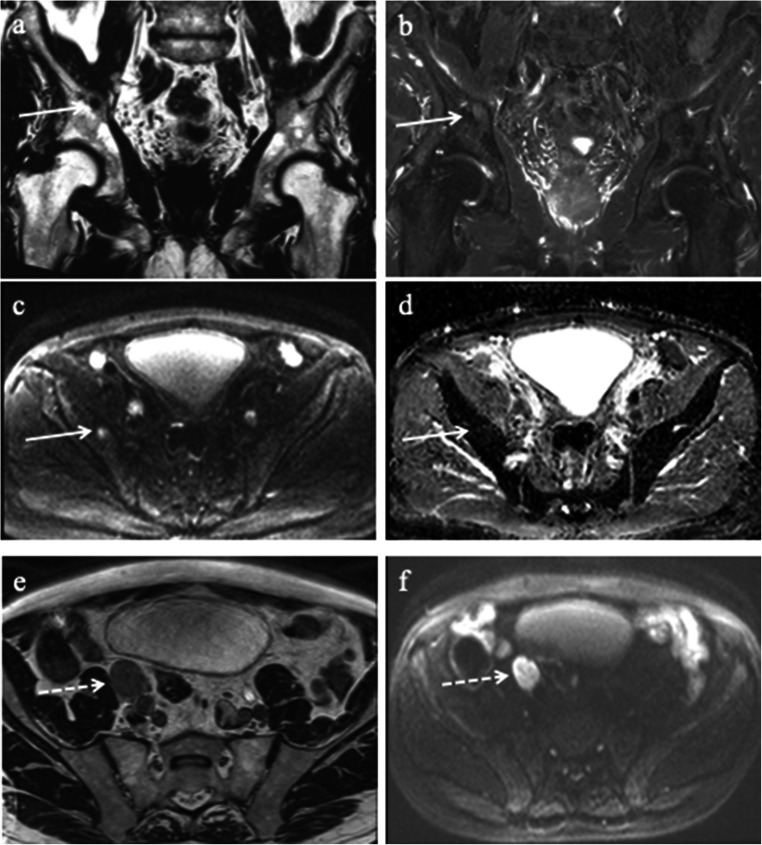
An 82-year-old patient with cT3/radT4, PSA 108 ng/ml, and ISUP 5. The solid arrow illustrates a metastasis in the right ischial body, and the stippled arrow indicates a regional lymph node metastasis. **a** Coronal T2_tse_dixon fat, (**b**) coronal T2_tse_dixon water, (**c**) axial DWI b800 s/mm^2^, (**d**) axial ADC map, (**e**) axial 3DT2, (**f**) axial DWI b800 s/mm^2^

Lymph node metastases were seen in 13% (95% CI: 10–16, 52 out of 390), of which 67% (95% CI: 54–78, 35 out of 52) were regional (Fig. 2), and 88% (95% CI: 77–95, 46 out of 52) had high-risk disease (Table 2). All patients with non-regional metastases had concomitant regional lymph node metastases. The median PSA in patients with- and without lymph node metastases was 24 ng/ml (IQR 13–92) and 11 ng/ml (IQR 8–17), respectively (*p* < 0.001). There was no difference in age. The median short axis of metastatic lymph nodes was 14 mm (IQR 9–20).

**Table 2:** The prevalence of metastases according to patient characteristics and risk-groups

	Total		Lymph node metastases		Bone metastases	
	*n*	%	*n*	%	*n*	%
**EAU risk group**						
Unfavorable intermediate risk	123	32	6	5	1	0.8
High-risk	267	68	46	17	28	10
Total	390	100	52	13	29	7
**ISUP grade**						
1	5	1	0	0	0	0
2	17	4	1	5	0	0
3	143	37	10	7	2	1
4	106	27	10	9	6	6
5	110	28	25	23	16	15
Missing	9	2	6	67	5	56
**Clinical T stage**						
1	120	31	7	6	2	2
2(a+b)	140	36	11	8	4	3
2c	7	2	1	14	1	14
3	66	17	20	30	14	23
4	10	3	6	60	2	20
Missing	47	12	7	15	6	13
**Radiological T stage**						
1	4	1	0	0	0	0
2	168	43	9	5	0	0
3a	120	31	10	8	7	6
3b	67	17	14	21	8	12
4	30	8	19	63	14	47
Missing	1	0	0	0	0	0

In the high-risk group, the rate of regional and non-regional metastases was 11% (95% CI: 7–15, 30 out of 267) and 6% (95% CI: 3–9, 16 out of 267), respectively. In the unfavorable intermediate-risk group, the rate of regional and non-regional metastases was 4% (95% CI: 1–9, 5 out of 123) and 0.8% (95% CI: 0–4, 1 out of 123), respectively.

RadT3-4 was associated with a 4-fold increased risk of lymph node metastases (Table 3). The risk was slightly higher for high-risk patients (OR 4.70, 95% CI: 1.9–11.6) while non-significant in the case of unfavorable intermediate-risk disease (OR 1.6, 95% CI: 0.3–8.3). Clinical T3-4 was associated with a 7-fold risk of lymph node metastases (Table 3), and lymph node metastases were associated with a 13 times higher risk of bone metastases (OR 13.2, 95% CI: 5.8–29.9)

**Table 3:** Rates and risk of metastases according to radiological and clinical T-stages

		Lymph node metastases					Bone metastases				
	Total	*n*	%	95% CI	OR	95% CI	*n*	%	95% CI	OR	95% CI
All patients**											
cT1-2	267	19	7	5–11	6.79	3.4–13.2	7	3	1–5	9.91	3.9–25.1
cT3-4	76	26	34	25–45			16	21	13–32		
RadT1-2	172	9	5	3–10	4.48	2.1–9.5	0	0	0–2	n.a*	n.a*
RadT3-4	217	43	20	15–26			29	13	9-19		

*Odds ratio cannot be calculated due to no cases of bone metastases in rad T1-2

**Missing cT in 47 patients and missing radT in one patient

### Bone metastases

Bone metastases were first suggested in 31 patients, of which one had equivocal findings deemed negative after a multidisciplinary team meeting. Three underwent bone biopsies, of which one was negative and two were positive. Consequently, the overall rate of metastases was 7% (95% CI: 5–10, 29 out of 390), and all were clinically managed as metastatic diseases (Table 2).

The rate of bone metastases in high-risk and unfavorable intermediate-risk patients was 10% (95% CI: 7–15, 28 out of 267) and 0.8% (95% CI: 0–4, 1 out of 123), respectively. The median PSA in patients with- and without metastases was 31 ng/ml (IQR 17–119) and 11 ng/ml (8–18), respectively (*p* < 0.001). There was no difference in age. The median size of the bone metastases was 20 mm (IQR 14–34), and ten patients had > 4 metastases, while 19 patients had ≤ 4.

When classified according to radT-stage, no patients with radT1-2 had bone metastases compared to 13% of those with radT3-4 (Table 3). When classified according to cT- stage, bone metastases were seen in 3% of those with cT1-2 and 21% of those with cT3-4 (OR 9.91).

**Fig. 3:**
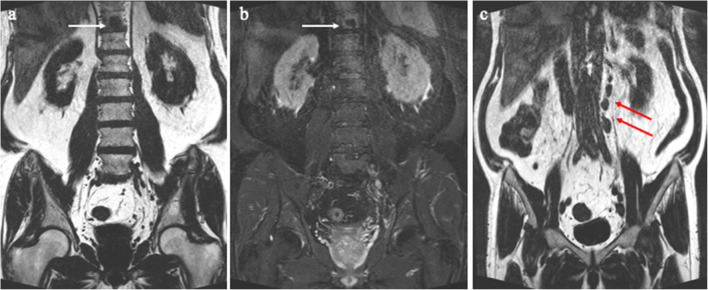
A 64-year-old patient with cT2/radT3a, PSA 39 ng/ml, and ISUP 5. The white arrow indicates a solitary metastasis in the 11^th^ thoracic vertebral body. The red arrows indicates multiple non-regional paraaortic lymph node metastases. In this case, the patient underwent a supplementary MRI of the complete column verifying an isolated bone metastasis. **a** Coronal T2_tse_dixon fat, **b** coronal T2_tse_dixon water, (**c**) coronal T2_tse_dixon fat

**Fig. 4:**
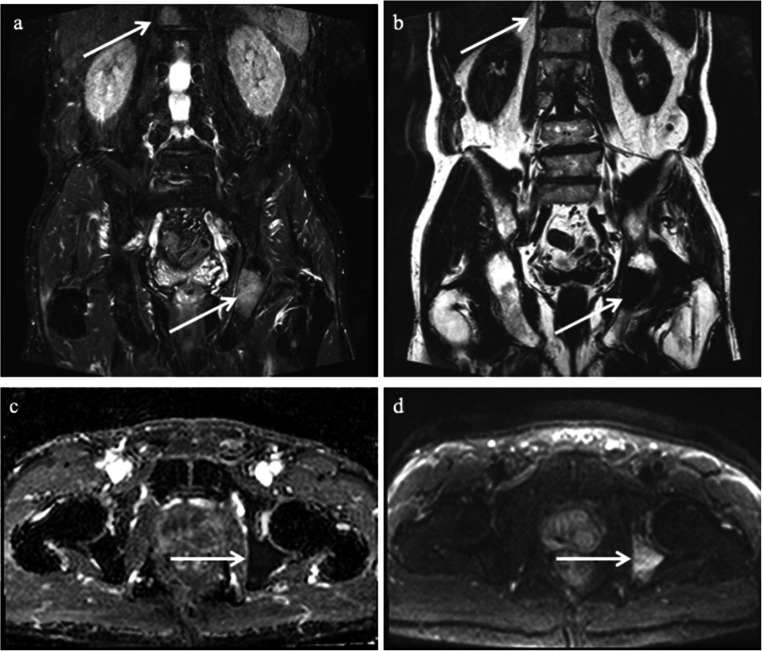
An 81-year-old patient with cT2/radT3a, PSA 9.5 ng/ml, and ISUP 3. The arrows indicate bone metastases in the left ischial body and the 12^th^ thoracic vertebral body. The prostate biopsies demonstrated a 90% Gleason grade 4 pattern in all ten biopsy cores. On reappraisal by a second uropathologist, this case was considered ISUP 4. **a** Coronal T2_tse_dixon water, (**b**) coronal T2_tse_dixon fat, (**c**) axial ADC map, (**d**) axial DWI b800 s/mm^2^

Metastases exclusively to the lumbar column occurred in 0.5% (95% CI: 0–2, 2 out of 390) (Fig. 3), exclusively to the pelvis in 4% (95% CI: 2–6, 14 out of 390), and both pelvis and lumbar column 3% (95% CI: 2–6, 13 out of 390) (Fig. 4).

## Discussion

Using an abbreviated MRI protocol of the lumbar column and pelvis, this study demonstrated bone metastases in 7% of those eligible for a metastatic workup according to the EAU guidelines. In addition, the rate of bone metastases was 10% in high-risk and 0.8% in case of unfavorable intermediate-risk. These rates are similar to a recent study using whole-body MRI and the same eligibility criteria [4].

The prevalence of bone metastases is highly dependent on patient selection and radiological methods, and updated studies reflecting contemporary practice are needed. A review from 2004 using BS stated that approximately 17% of all patients had bone metastases at the time of diagnosis, and 49% in those with locally advanced disease [30]. More recent MRI studies reported 1.5–6.8% metastases in newly diagnosed prostate cancer [5, 27]. However, those studies described neither risk groups nor patient characteristics in detail, and comparison to our study is difficult. The dramatic decrease in the prevalence of bone metastases is probably due to stage migration caused by improved diagnostics and the widespread use of PSA.

We found a marginal rate of bone metastases (0.8%) in the unfavorable intermediate-risk group, comparable to the 0–1% found in previous studies with similar patient groups [4, 31, 32]. The studies in the EAU guidelines found 13–22% positive BSs in patients with a Gleason score of 7, but the PSA and cT-stage in those patients were not reported [33, 34]. Furthermore, they assigned patients to risk groups deviating from current practice, making a comparison with the unfavorable intermediate-risk group impossible. According to modern MRI studies, the prevalence of bone metastases in the unfavorable intermediate-risk group is very low, and the need for metastatic workup should be reconsidered.

In this study, two high-risk patients (0.5%) had metastases in the column without concomitant metastases in the pelvis (Fig. 3). The overall low rate of metastases exclusively to the column is in accordance with earlier studies (0–0.3%), supporting an initial MRI limited to the pelvic-lumbar region [4, 5].

Our abbreviated MRI protocol for bone metastases combined DWI and T2 Dixon of the pelvis and T2 Dixon of the lumbar column. T2 Dixon imaging is time-efficient since one acquisition generates “fat-only” and “water only” images, similar to the traditional T1W and STIR [26, 28]. Larbi et al showed that adding DWI to T1w+STIR did not improve the detection of bone metastases [28]. Still, other studies report that DWI has an added value in differentiating benign degenerative lesions and metastases, thus reducing the false-positive rate [28, 35–37]. Therefore, we believe it is essential to assess the ADC map with the other images. In general, metastases usually demonstrate low diffusion compared to benign lesions, but no specific cut-off values can be applied due to overlapping values of normal marrow and metastases [38]. Untreated metastases usually have the same or slightly higher ADC than normal marrow, as seen in Figs. 2 and 4.

Fat detection in bone marrow lesions is crucial since untreated metastases usually do not contain fat [27]. However, fat content is subject to individual judgment, and there might be borderline cases. The optimal fat fraction for distinguishing benign and malignant vertebral fractures ranges from 5 to 11% [36, 39, 40]. However, it is unknown how fat-fractioning will perform in patients without fractures. Only two studies have compared PSMA-PET/CT and MRI for detecting bone metastases [41, 42]. These studies conclude that PSMA-PET/CT is superior to MRI, but sample size and eligibility criteria limit the generalizability. In one of the studies (*n* = 55), only 18% were in the primary setting, while the majority were under androgen deprivation therapy (ADT) [41]. In the second study (*n* = 68), all patients had PSA recurrence after primary treatment [42]. The diagnostic criteria for bone metastases are different in the case of treatment naïve patients, PSA recurrence, or ADT response evaluation. Notably, whereas fat is considered a benign sign in treatment-naïve patients, it may be a sign of treatment response in the case of ADT. The different populations make it difficult to assess whether PSMA-PET/CT is superior to MRI in the primary setting, and specific studies are needed.

In this study, all bone metastases were found in patients with radT3-4. In comparison, bone metastases occurred in all clinical T-stages. Hence, radT-stage was a better predictor for bone metastases than cT-stage, and potentially, one could omit a metastatic workup in patients with radT1-2. MRI upgraded 47% of patients classified as cT1-2 and downgraded 13% of those classified as cT3-4. Although we cannot provide a prostatectomy specimen as the reference standard, previous studies have demonstrated that MRI is far more accurate than digital rectal examination for local staging [22, 43–46]. The relatively high rate of missing cT-data (12%) may affect results. To our knowledge, no prior studies have examined the association between radT-stage and bone metastases in treatment -naïve patients. Although radT-staging is challenging, it may add valuable information to the preoperative risk nomograms.

We detected lymph node metastases in 13%, of which 88% had high-risk disease. Radiological T3-4 increased the risk of lymph node metastases by 4.5, while cT3-4 increased the risk by 6.8. To the best of our knowledge, no other studies have assessed the correlation between radT- and the risk of lymph node metastases. We used morphologic and functional criteria for pelvic lymph node metastases based on T2w and DWI. Metastatic lymph nodes usually demonstrate low diffusion, i.e., high signal on high *b*-value images and low signal on the ADC map. However, due to a significant overlap in ADC values between benign and metastatic lymph nodes, Thoeny et al suggested not using specific cut-off values but primarily assessing the high *b*-value images [24, 47]. We also used T2Dixon to detect enlarged retroperitoneal lymph nodes.

Nevertheless, no criteria can accurately differentiate small metastases from benign lymph nodes, and the sensitivity of MRI for detecting lymph node metastases is generally low. Although PSMA is considered more sensitive, any radiological assessment of lymph node metastases is inadequate [11–13]. Therefore, pelvic lymph node dissection continues to be the gold standard for staging [1].

The main limitation of this study is due to the predominantly descriptive reference standards as only a few patients underwent bone biopsies, and histology results from lymph nodes are lacking. We used no a priori defined size criteria and no rigid cut-off values for determining fat percentage in bone metastases. Therefore, we may have underreported small lesions sensitive to partial volume effects. MRI acquisition and reporting were not according to the MET-RADS standard, and we did not perform a standardized follow-up in negative patients [29]. Some patients had missing data, especially clinical T-stage, and some did not undergo a prostate biopsy due to comorbidity.

We consider an abbreviated MRI consisting of T2 Dixon with matching DWI of the lumbar column and pelvis to be well suited for a “one-stop-shop” approach when staging treatment naïve prostate cancer. One could also replace the 3DT2w images with a 3–5-mm 2D T2w images to speed up image acquisition. This strategy would be more accurate and significantly faster than a 3–4-hours BS combined with abdominal cross-sectional images.

In conclusion, the overall prevalence of bone metastases was 10% in high-risk patients and 0.8% in case of unfavorable intermediate-risk. The low prevalence of metastases in the unfavorable intermediate-risk group suggests it is redundant to screen these patients. Furthermore, we did not find any bone metastases in patients with radT1-2 disease, indicating that radT classification may improve the prediction of bone metastases.

## Abbreviations

BS: Bone scan
cT: Clinical T-stage
CT: Computed tomography
EAU: European Association of Urology
ISUP: International Society of Urogenital Pathology
MRI: Magnetic resonance imaging
PET: Positron emission tomography
PSA: Prostate-specific antigen
PSMA: Prostate-specific membrane antigen
radT: Radiological T-stage

## Declarations

### Guarantor

The scientific guarantor of this publication is Erik Rud.

### Statistics and biometry

No complex statistical methods were necessary for this paper.

### Methodology

• Retrospective

• diagnostic or prognostic study

• performed at one institution
